# Regulation of Neutrophil Degranulation and Cytokine Secretion: A Novel Model Approach Based on Linear Fitting

**DOI:** 10.1155/2015/817038

**Published:** 2015-10-22

**Authors:** Isabelle Naegelen, Nicolas Beaume, Sébastien Plançon, Véronique Schenten, Eric J. Tschirhart, Sabrina Bréchard

**Affiliations:** Calcium Signalling and Inflammation, Life Sciences Research Unit, University of Luxembourg, 162A Avenue de la Faïencerie, 1511 Luxembourg, Luxembourg

## Abstract

Neutrophils participate in the maintenance of host integrity by releasing various cytotoxic proteins during degranulation. Due to recent advances, a major role has been attributed to neutrophil-derived cytokine secretion in the initiation, exacerbation, and resolution of inflammatory responses. Because the release of neutrophil-derived products orchestrates the action of other immune cells at the infection site and, thus, can contribute to the development of chronic inflammatory diseases, we aimed to investigate in more detail the spatiotemporal regulation of neutrophil-mediated release mechanisms of proinflammatory mediators. Purified human neutrophils were stimulated for different time points with lipopolysaccharide. Cells and supernatants were analyzed by flow cytometry techniques and used to establish secretion profiles of granules and cytokines. To analyze the link between cytokine release and degranulation time series, we propose an original strategy based on linear fitting, which may be used as a guideline, to (i) define the relationship of granule proteins and cytokines secreted to the inflammatory site and (ii) investigate the spatial regulation of neutrophil cytokine release. The model approach presented here aims to predict the correlation between neutrophil-derived cytokine secretion and degranulation and may easily be extrapolated to investigate the relationship between other types of time series of functional processes.

## 1. Introduction

Historically, neutrophils were described as simple professional killers of invading pathogens to the human organism [1]. In this regard, it was considered that only the release of various antimicrobial and cytotoxic proteins synthesized and distributed into different types of granules participated to the innate immune response mediated by neutrophils. Granule types have been characterized to be readily mobilized upon an inflammatory stimulus at the plasma membrane in reverse order to their formation according to the* formed-first-released-last model* [2]. Indeed, in the different stages of neutrophil development, azurophil granules are formed first followed by specific granules, gelatinase granules, and, lastly, secretory vesicles, which are the most easily mobilized organelles in the mature neutrophils.

Due to recent progress, this classical view has been expanded by the acknowledgment that appropriately activated neutrophils constitute a substantial source of a variety of secreted cytokines supporting a direct contribution of these cells in the regulation framework of the adaptive immune response [3–5]. Neutrophils not only are a source of* de novo* synthesized cytokines dependent on gene induction but also have the capacity to express cytokines at a basal level from preformed stores [2]. However, precise intracellular localization of these packaged cytokines and mechanisms underlining their secretion remain largely elusive. The widely accepted assumption is that multiple secretory pathways coexist in neutrophils allowing the regulated release of diverse proinflammatory mediators [6]. Preformed cytokines are instantly released upon ligand-receptor signaling during the so-called “regulated exocytosis” process [7] whereas* de novo* synthesized cytokines may be released after trafficking via recycling endosomes during the mechanism referred to as “constitutive exocytosis” [8, 9]. Additionally, variations of these two main classical secretion pathways have also been reported [10]. These distinct processes selectively control the combination of granule proteins and cytokines released into the local microenvironment from neutrophils over a temporal and spatial range and are thus regulatory mechanisms important for the onset and resolution of inflammation enabling the development of an appropriate inflammatory response [11].

It is now largely recognized that neutrophil-derived granule proteins and cytokines contribute to the maintenance of the inflammatory response and, when excessively secreted, to the ongoing process of tissue damage leading to the development of many chronic inflammatory disorders such as inflammatory bowel diseases [12], rheumatoid arthritis [13], chronic obstructive pulmonary disease [14], and atherosclerosis [15]. Determination of the regulatory mechanisms mediating the different patterns of cytokine trafficking and release may create opportunities to define new targets or strategies to selectively reduce cytokine secretion in clinical diseases.

Therefore, we selected relevant cytokines secreted by neutrophils, described to contribute to the development of chronic inflammatory diseases, in order to investigate their release in combination to degranulation upon stimulation with bacterial lipopolysaccharide (LPS).

Here, we propose an appealing model based on a linear fitting approach of cytokine secretion and degranulation giving a first basis for deeper understanding of the relationship between these two processes. It also provides a predictive view on the distribution of cytokines in neutrophils and offers an outstanding starting point to target future research on release mechanisms involved in inflammatory processes.

## 2. Materials and Methods

### 2.1. Purification of Human Neutrophils

Peripheral blood of healthy volunteers was collected in EDTA-containing tubes (BD Vacutainer, BD Biosciences, Erembodegem, Belgium). Samples were collected in accordance with the good clinical and ethical practices, which have been approved by the Ethics Review Panel (ERP) of the University of Luxembourg according to the “Comité National d'Ethique de Recherche” (CNER) from Luxembourg.

Neutrophils were isolated from blood samples by Polymorphprep separation procedure (Axis-Shield, Dundee, Scotland) according to manufacturer's instructions. Remaining erythrocytes in the neutrophil cell suspension were lysed for 10 min with red blood cell lysis buffer (155 mM NH_4_Cl, 10 mM KHCO_3_, 0.1 mM EDTA, and pH 7.4 [16]).

Neutrophils were washed and resuspended in PBS 1x (pH 7.4). Purity of isolated neutrophils was analyzed by the BD FACSCanto II flow cytometer (BD Biosciences) using two mixtures of selection markers CD66b-FITC/CD11b-PE/CD14-APC and CD15-FITC/CD16-PE/CD45-APC (Immunotools, Friesoythe, Germany) on 10,000 events in the gated population of homogenous (FSC-A versus SSC-A), single (SSC-A versus SSC-H), and living cells (negative cells for Sytox Blue staining (Invitrogen, Gent, Belgium)). Purified neutrophils are positive for all the selection markers used by flow cytometry. Human neutrophils were cultured in X-VIVO 15 medium with L-glutamine and gentamicin (Lonza) at 37°C and 5% CO_2_ up to 24 h after purification.

### 2.2. Cell Stimulation

Purified neutrophils were stimulated with bacterial LPS from* E. coli* serotype O111:B4 (Sigma, Bornem, Belgium) for simulating proinflammatory conditions. For kinetic studies of cytokine secretion and degranulation, neutrophils were stimulated with 100 ng/mL LPS for 2, 4, 6, 12, and 24 h under serum-free conditions to avoid any serum component contamination, which could interfere with specific LPS-induced cell responses.

### 2.3. Cell Analysis by Flow Cytometry

In accordance with the literature, the most relevant markers have been selected for degranulation analysis [17]. Degranulation was determined by measuring the expression of CD markers characteristic for azurophil granules (CD63-PE), specific granules (CD15-FITC, CD66b-FITC), gelatinase granules (CD11b-PE), and secretory vesicles (CD13-APC, CD14-APC, CD18-FITC, and CD45-APC) at the plasma membrane by flow cytometry (all antibodies are from BD Biosciences except CD14-APC from Immunotools).

IgG1-FITC, IgG2a-PE (BD Biosciences), and IgG1-APC antibodies (Immunotools) were used as negative isotype controls to place the cells in the first decade of any plot, whereas CD45-FITC, CD45-PE, or CD45-APC (BD Biosciences) single dye staining was used to set compensations. Data analysis was performed by measuring the mean fluorescence intensity (MFI) for each CD marker with BD FACSDiva software (BD Biosciences) on the gated population of granulocytes (FSC-A versus SSC-A), single (SSC-A versus SSC-H), and living cells (negative cells for Sytox Blue staining (Invitrogen)). In total, 10,000 events were recorded* per* staining. The relative translocation of CD markers to the plasma membrane for each granule was determined by calculating the ratio between MFI of LPS-stimulated cells and nonstimulated control from the same time point.

### 2.4. Measurement of Cytokine Secretion by Cytometric Bead Array (CBA)

Density of human neutrophils was adjusted to 10 × 10^6^ cells* per* condition for subsequent quantitative measurement of cytokine secretion by LPS-stimulated cells, respectively. Fresh supernatants were collected and used directly for cytometric bead array (CBA, BD Biosciences) analysis. The multiplex standard curve composed of mixed cytokine standards was set up by serial dilutions according to the manufacturer's instructions. Selected capture beads were prepared and added to supernatants. The following beads were used: CCL2 (MCP1, bead D8), CCL3 (MIP1*α*, bead B9), CCL4 (MIP1*β*, bead E4), CCL5 (RANTES, bead D4), IL1a (bead D6), IL1b (bead B4), IL6 (bead A7), IL8 (CXCL8, bead A9), IL12b (bead E5), and TNFa (bead C4). After 1 h of incubation, detection reagent was added to each sample. After 2 h of incubation, samples were rinsed with wash buffer and centrifuged. Samples were washed again prior to flow cytometry analysis (BD FACSCanto II, BD Biosciences). Results were quantified using the standard curves and the Flow Cytometric Analysis Program (FCAP) Array software (Soft Flow, Minneapolis, USA).

### 2.5. Linear Fitting Approach via R Statistical Software

Kinetic profiles of cytokine secretion and degranulation were imported into R statistical software (https://www.r-project.org/) and a linear regression approach was applied. This approach was used to find the optimal proportionality factor, namely, the slope of the model, and provide methods to evaluate the significance of our models. All ratio values between LPS-stimulated and nonstimulated control conditions (stimulation points 0, 2, 4, 6, 12, and 24 h) from the time series of cytokine secretion and degranulation were log_10_ normalized to minimize scale effect. For each granule-specific CD marker, a linear model has been fitted with the secreted cytokines. For each model, ANOVA analysis has been performed and the adjusted *R*-squared (RSQ) value and the slope of the model were retained. Models with a significant difference to the null model (*p* value ≤ 0.05) and with a high adjusted RSQ value between degranulation marker and secreted cytokine (RSQ ≥ *r*, with *r* being determined by simulations; see Section 2) were considered as underlying a strong similarity of pattern between a secreted cytokine and a mobilized degranulation marker. Then, cytokines linked to the same degranulation marker were clustered. To visualize these clusters, time series of the degranulation marker and its relative cytokines of the cluster were plotted. To permit comparison between time series from different scales, values from the secreted cytokines were divided by the slope of their linear model.

### 2.6. Simulations to Determine the Optimal RSQ Threshold

To define the optimal RSQ threshold, we simulated the fitting between pairs of granule marker and cytokine with controlled perturbations between them and choose the threshold by determining at which level of perturbation the RSQ was drastically dropping. The simulations were designed as follows: time series of each granule-specific marker and cytokines were taken individually (19 time series in total) and used to simulate matching time series with more or less perturbations. For the six points of each time series (0, 2, 4, 6, 12, and 24 h), a random number between *V*_*i*_ − *e* and *V*_*i*_ + *e* was drawn. *V*_*i*_ is the *i*th element of the time series and *e* is a predefined constant. *e* controls the intensity of the perturbation: the highest *e* is the more different both profiles are expected to be. All values from 0 to 1 with a step of 0.1 were tested for *e* (0, 0.1, 0.2, etc., to 1). Then, the RSQ of the original profile versus simulated profile was computed as mentioned in* linear fitting approach via R statistical software*. This process was repeated 1000 times and, for each of the 19 original profiles, the average of the RSQs was computed and plotted against the *e* value. To define the optimal RSQ threshold that defines a cut-off between linear fitting models with “high RSQ” and “low RSQ,” we clustered the distribution of averaged RSQ into two groups, using a *k*-means approach (a silhouette analysis of all clustering solution with 2 to 10 clusters confirmed that using 2 clusters was the best solution,* data not shown*). The last element of the cluster with the highest center, namely, 0.796, was taken as RSQ threshold for our analysis.

### 2.7. Statistical Analysis

Statistical analyses were performed using the PRISM6 software (Graph Pad Software, La Jolla, CA, USA). When normality and homogeneity of variances were ascertained, as determined by the *F*-test, Student's *t*-test analyses were performed to establish two group comparisons. Otherwise, Mann-Whitney tests were used for two group comparisons. *p* values ≤ 0.05 were considered statistically significant.

## 3. Results

### 3.1. Cytokine Levels Secreted by Human Neutrophils upon LPS Stimulation

Since recent reports have implicated neutrophils in the development of chronic inflammatory disorders, we wanted to characterize the regulatory mechanisms in the release of neutrophil-derived products. In a first step, cytokine candidates found secreted by highly purified (≥98%) neutrophils upon LPS stimulation [17] were selected for integration into our mathematical model. These cytokines have a particular relevance since they have been reported to contribute to the development of different chronic inflammatory diseases through the recruitment of diverse immune cells to the inflammatory site (Table 1).

**Figure 1:**
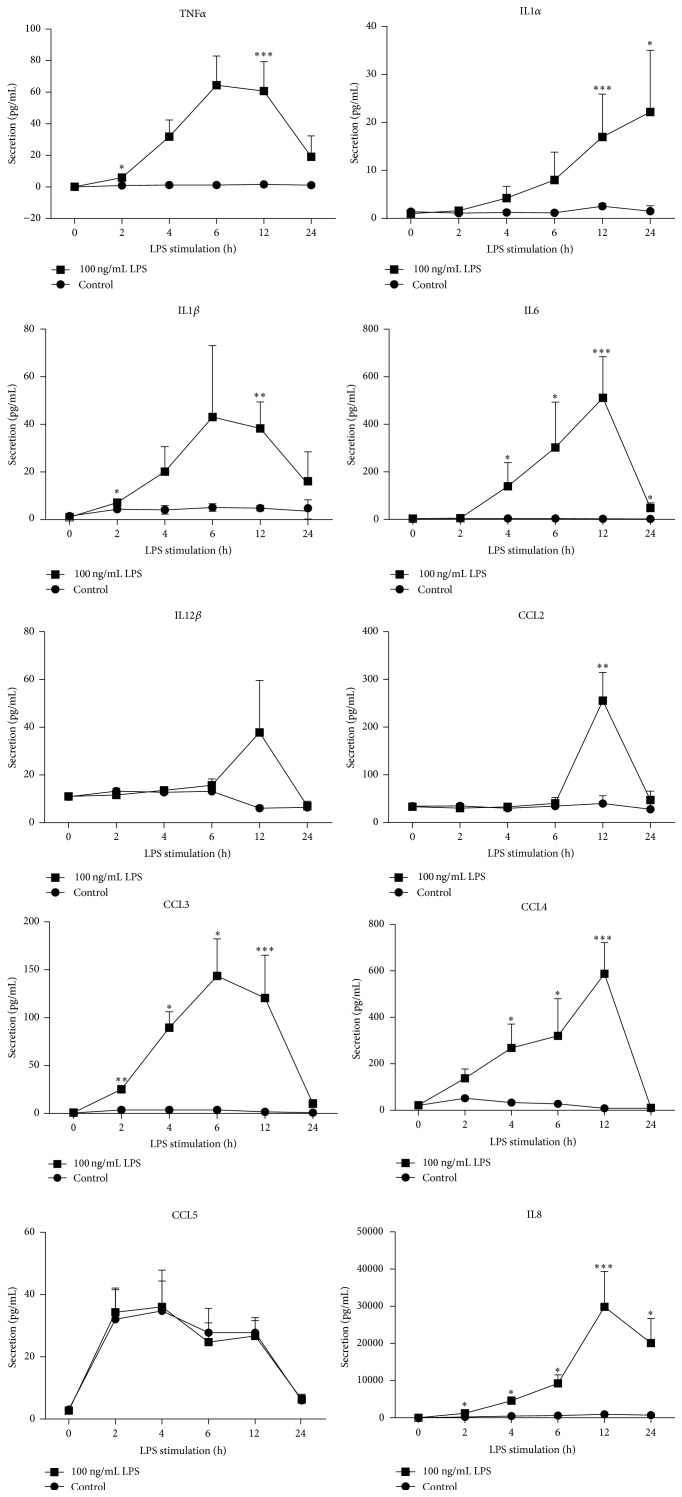
Time-dependent effect of LPS on cytokine secretion in human neutrophils. Cytokine secretion was measured by CBA upon stimulation with 100 ng/mL LPS for 0 h, 2 h, 4 h, 6 h, 12 h, and 24 h. Results are mean secretion (pg/mL) ± SEM of at least 3 independent experiments, significantly different from cytokine secretion in nonstimulated control at the corresponding time: ^*∗*^ *p* < 0.05, ^*∗∗*^ *p* < 0.01, ^*∗∗∗*^ *p* < 0.001, and ^*∗∗∗∗*^ *p* < 0.0001.

To develop a reliable model that investigates the relationship between degranulation and cytokine secretion, different experimental data sets were generated. Time series of cytokine secretion were determined to serve as input to our model. Neutrophils were treated for 0 h, 2 h, 4 h, 6 h, 12 h, and 24 h with LPS 100 ng/mL since maximal peak of secretion for cytokines was reached at this concentration (data not shown). Subsequent quantitative measurement of cytokine secretion was performed by the CBA technique. A basal secretion level of all cytokines was detected in supernatants from neutrophils under nonstimulated conditions. Secretion of most of the cytokines released into the extracellular medium was augmented with increasing time of LPS stimulation, except for IL12*β* and CCL5 whose release was not significantly affected by LPS treatment (Figure 1).

The secretion pattern was different for each cytokine in the way that different profiles could be identified. Except for IL1*α*, secretion levels of cytokines were maximal 6 h or 12 h after treatment with LPS and decreased after 24 h. Maximal cytokine secretion was observed at (i) 6 h LPS for CCL3 and (ii) 12 h LPS for TNF*α*, IL6, IL8, CCL2, IL1*β*, and CCL4. Moreover, quantities of released cytokines were highly variable. TNF*α*, IL1*α*, IL1*β*, and CCL3 were only discretely secreted (≤150 pg/mL) whereas IL6, CCL2, and CCL4 were secreted at an intermediate level (~250–600 pg/mL) and IL8 was highly secreted (≥30000 pg/mL).

### 3.2. Effect of LPS on Degranulation in Neutrophils

To collect data for the implementation of the model, the second series of experiments consisted in identifying the kinetic degranulation profile of the different granule types upon time-dependent LPS stimulation. Degranulation can be determined by the upregulation of granule membrane molecules as a consequence of membrane fusion from granules with the plasma membrane [35]. Therefore, LPS-treated cells were analyzed for cell surface expression of several CD molecules known as degranulation markers.

**Figure 2:**
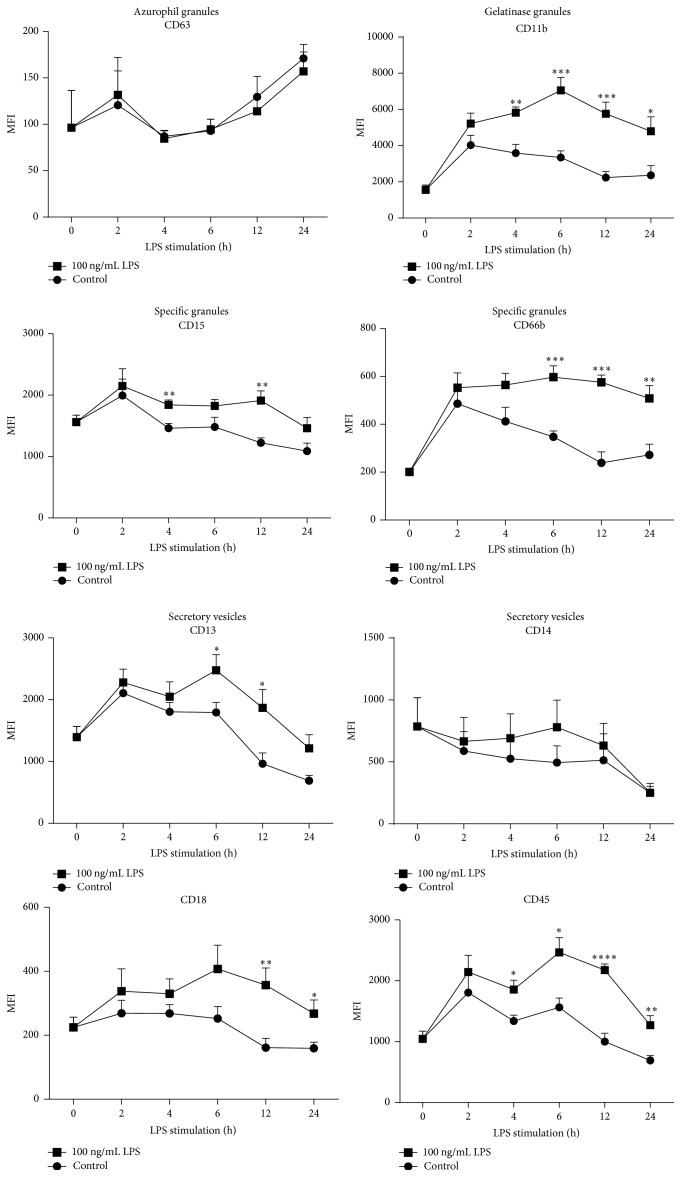
Temporal pattern of degranulation upon LPS stimulation in human neutrophils. Translocation of degranulation markers to the plasma membrane was assessed using flow cytometry after cell treatment with 100 ng/mL LPS for 0 h, 2 h, 4 h, 6 h, 12 h, and 24 h. Sytox Blue negative cells were gated to exclude dead cells from the analysis. Results are expressed in mean fluorescence intensity (MFI) of LPS-stimulated cells and nonstimulated control ± SEM of at least 3 independent experiments, significantly different from nonstimulated control at the same time point: ^*∗*^ *p* < 0.05, ^*∗∗*^ *p* < 0.01, ^*∗∗∗*^ *p* < 0.001, and ^*∗∗∗∗*^ *p* < 0.0001.

Results showed that LPS stimulation affected the ease of mobilization of intracellular granule types in neutrophils in a time-dependent manner, as reflected by increased translocation of CD markers to the cell surface (Figure 2). In a temporal pattern, LPS stimulation induced the release of specific granules as demonstrated by the redistribution of CD15 and CD66b to the plasma membrane. Translocation of these CD markers towards the plasma membrane was scattered over a time interval of 4 h and 24 h.

In a similar way, LPS stimulation increased the presence of CD11b as well as CD13, CD18, and CD45 at the plasma membrane reflecting an increased release of gelatinase granules and secretory vesicles, respectively. As observed with CD15 and CD66b for the specific granules, increase of these CD markers was detected between 4 h and 24 h of LPS stimulation.

Maximal expression at the plasma membrane for all the CD markers was detected after 6 h or 12 h of LPS stimulation (Figure 2).

It must be noted that LPS was unable to trigger the mobilization of azurophil granules since CD63 expression was not changed at the plasma membrane.

### 3.3. Linear Fitting of Cytokine Secretion and Degranulation Kinetics

**Figure 3:**
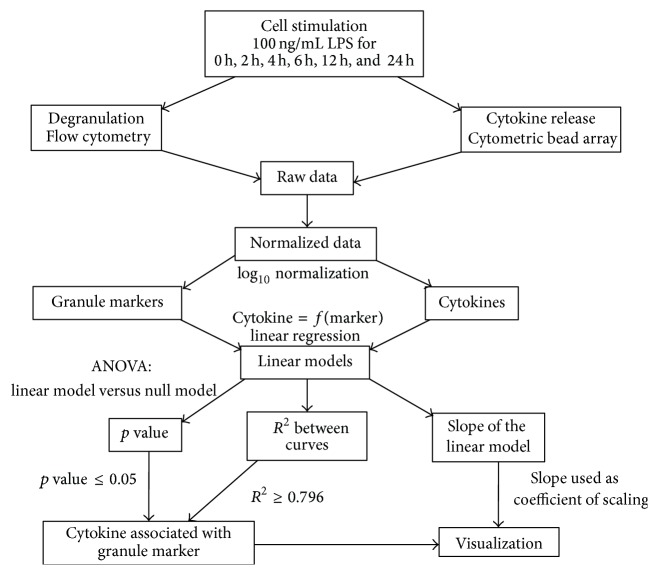
Workflow of linear fitting of LPS-mediated cytokine secretion and degranulation. A novel approach based on linear fitting was used to find linear relationship between short-time series of secreted cytokines and similar pattern to degranulation markers present at the plasma membrane. After importing data into R statistical software, all ratio values between LPS-stimulated and nonstimulated control conditions were log_10_ normalized and treated as mentioned in Section 2.

Many approaches exist for the examination of time series of expression data (e.g., [36]) but none of them could be applied to analyze short-time series of secretion. For this reason, we used a novel model approach to explore the relationship between cytokine secretion and degranulation by their kinetic profiles (Figure 3).

We hypothesized that time series of secreted cytokines with similar pattern to time series of degranulation markers present at the plasma membrane should have proportional values at each time point of LPS stimulation, so that a proportionality factor between the two profile curves can be defined. To address this question, we choose to use the linear regression approach, which fits best to our needs: it captures proportionality well, can be used with only one pair of profiles (in our case, cytokine versus granule-specific marker), and includes measures to evaluate the results (significance of the model and *R*-squared value, Section 2). All ratio values between LPS-stimulated and nonstimulated control conditions from the time series of cytokine secretion and degranulation (Table 2) were log_10_ normalized.

While the ANOVA analyses the efficiency of this model (i.e., proportional kinetic profile curves are significantly different from the null model), the adjusted RSQ value measures the correlation between the kinetic profiles (i.e., proximity to the linear fitting). The optimal RSQ value was determined by simulations, in which predefined perturbations were introduced to our kinetic profiles (Section 2).

**Figure 4:**
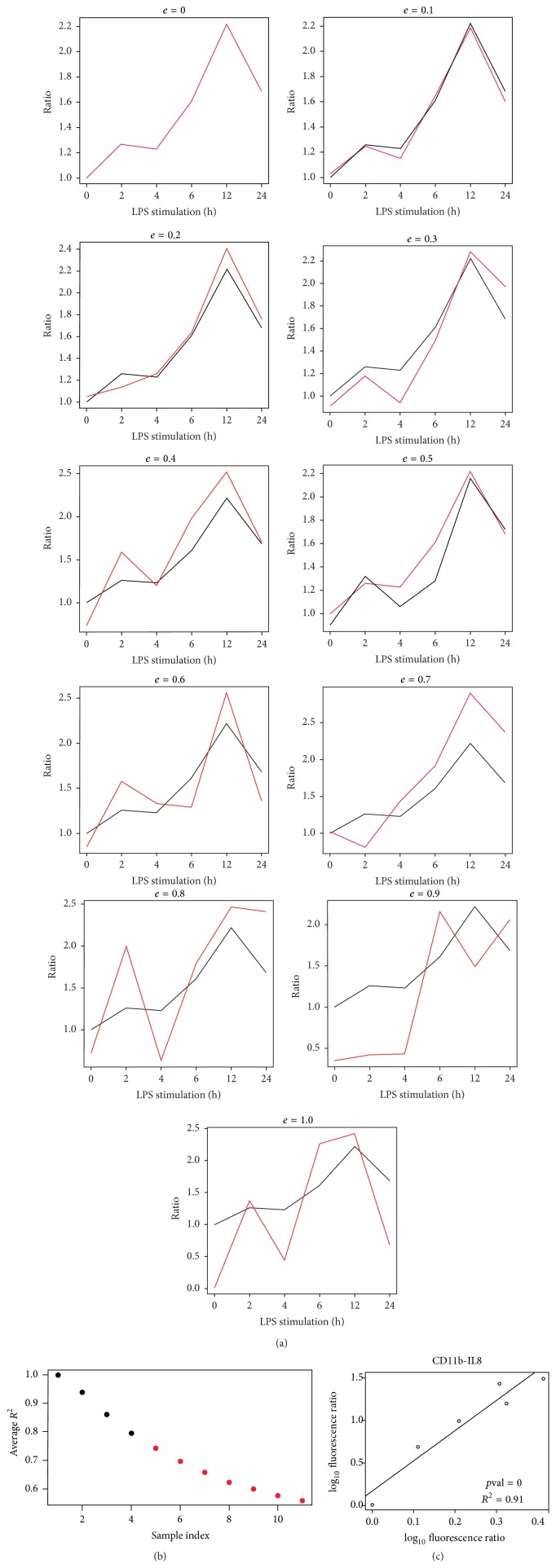
Linear fitting approach to investigate time series of degranulation and cytokine secretion. (a) Simulations were performed to find the optimal threshold for RSQ value. After introducing perturbations (*e* value) to the kinetic profiles, the linear fitting decreases in significance. (b) Plot of average RSQ deriving from simulations against augmenting *e* values. (c) Perfectly linear fitting model in which the behaviour of IL8 is correlated to CD11b. Due to its *p* value of 0 and RSQ of 0.91, the correlation between IL8 and CD11b corresponds to a perfect linear fitting model.

How augmenting perturbations (*e* from 0 to 1) influenced the linear fitting of two time series, in our example, the linear fitting between the granule marker CD11b and the cytokine IL8, is presented in Figure 4(a). The original kinetic profile is depicted by the black line whereas the one with perturbations is represented by the red line. By plotting the average RSQ values (derived from 1000 repetitions of simulations) against *e* values, *k*-means clustering can differentiate between “high” (black) and “low” (red) RSQ (Figure 4(b)).

By setting these parameters nonsignificant outcomes with “low” RSQ were eliminated, and the threshold for RSQ was set to RSQ ≥ 0.796.

An example of a significant linear fitting model is shown (Figure 4(c)), in which the behaviour of IL8 is correlated to CD11b. Due to its *p* value of 0 and RSQ of 0.91, the correlation between IL8 and CD11b fits to the model.

### 3.4. Relationship between Degranulation and Cytokine Secretion

**Figure 5:**
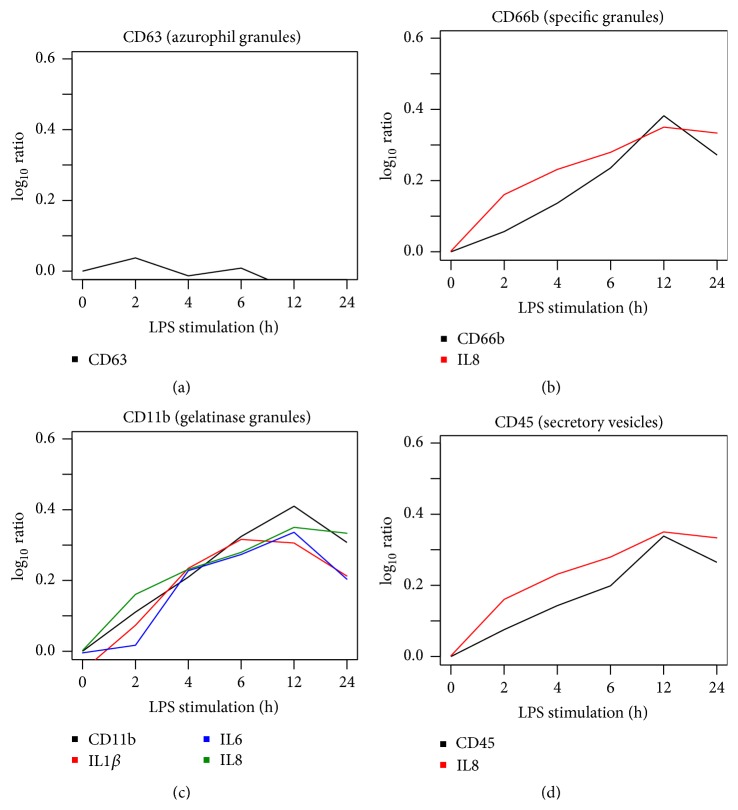
Linear fitting of LPS-mediated kinetics of cytokine release and degranulation in human neutrophils. Enlisted cytokines on the histograms fitted to the kinetics of the degranulation markers characteristic for (a) azurophil granules (CD63), (b) specific granules (CD66b), (c) gelatinase granules (CD11b), and (d) secretory vesicles (CD45), according to the selection of *p* value ≤ 0.05 and RSQ ≥ 0.82.

After filtering only highly significant correlation data, results from human neutrophils were plotted. The granule membrane molecules (CD63, CD66b, CD11b, and CD45), specific of each type of granule, that are most highly upregulated have been targeted to show the linear fitting approach (Figure 5).

This method applied to the time series data showed that the secretion of three selected proinflammatory cytokines (IL8, IL6, and IL1*β*) strongly correlated with the release of secretory vesicles, gelatinase granules, and specific granules (Figure 5).

The release of the cytokine IL8 fitted to CD66b suggesting that secretion of this cytokine correlated to specific granules (Figure 5(b)). Moreover, time series of IL1*β*, IL8, and IL6 release were strongly correlated to the degranulation marker CD11b, showing a relationship between gelatinase granules and these cytokines (Figure 5(c)).

Furthermore, secretory vesicles represented by the marker CD45 were fitted to IL8 (Figure 5(d)).

Since no significant cytokine correlation has been observed for CD63, azurophil granules are probably not associated with cytokine secretion (Figure 5(a)).

## 4. Discussion

For many years, the contributory impact of neutrophils to the development of chronic inflammation was not seriously taken into account since they have been considered as terminally differentiated cells synthesizing low amount of RNA and protein [37]. However, the vast number of neutrophils found at the site of infection cannot be neglected due to the fact that their secreted amounts of granule proteins and cytokines exert a cumulative and synergistic effect on the inflammatory tissue environment [15]. These proinflammatory soluble mediators are highly decisive for the onset of inflammatory processes and the activation and the recruitment of various immune cells to the infection site [1]. However, little is known about the combination in which cytokines and granule proteins are secreted by neutrophils. In the present report, we therefore aimed to predict the spatiotemporal regulation of proinflammatory mediator release in neutrophils. For this purpose, LPS has been used as stimulus agent since it has been well described to induce the secretion of granule contents and cytokines [17, 38]. Once the model is established, it could constitute an important tool to investigate other stimulation conditions (fMLF, TNF*α*, IL8, or combination of stimuli) in order to mimic different microenvironmental conditions (e.g., healthy and pathological diseases) and help to improve our knowledge of inflammatory processes.

**Figure 6:**
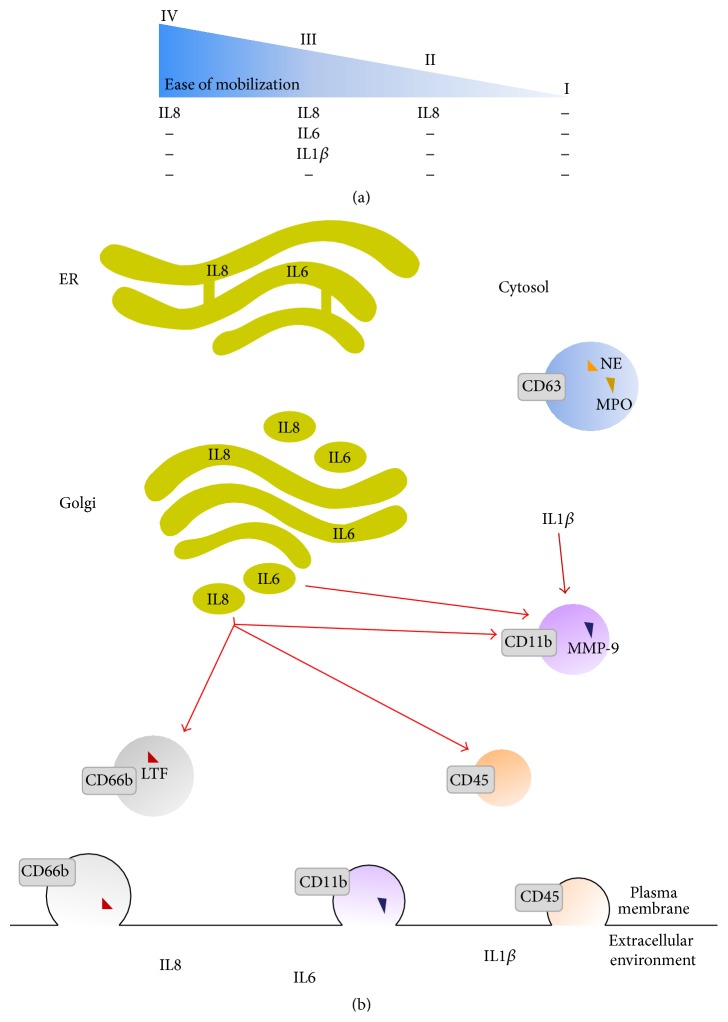
Hypothetical models of fitted cytokines and granules in human neutrophils. (a) Neutrophil-derived cytokines are released in a hierarchical sequence coincident with the roles of granules (IV secretory vesicles, III gelatinase granules, II specific granules, and I azurophil granules) during the microbial elimination processes and inflammatory response. (b) Cytokines and granules are released in a concurrent fashion but could additionally be localized in or mobilized to the different granule types. Classical secretory pathways are mediated through the endoplasmic reticulum (ER) and Golgi complex (IL6 and IL8). IL1*β* is secreted on a nonconventional pathway. Possible routes for cytokine trafficking (after ER-Golgi or after cleavage) and mobilization to the plasma membrane relative to degranulation are shown in red. Different types of granules are characterized by their CD markers and proteolytic enzymes (triangles): CD63, myeloperoxidase (MPO), and neutrophil elastase (NE) for azurophil granules; CD66b and lactoferrin (LTF) for specific granules; CD11b and matrix metallopeptidase-9 (MMP-9) for gelatinase granules; and CD45 for secretory vesicles. Upon mobilization of the granules to the plasma membrane, granule docking and fusion lead to the translocation of the CD markers to the plasma membrane and the release of proteolytic enzymes.

Our study is the first to propose an original approach allowing the establishment of a relationship between cytokine secretion and degranulation in neutrophils. We choose to use the linear fitting approach to integrate data generated from own experiments and obtained from LPS-mediated short-time series of degranulation and cytokine secretion. Other approaches, such as Pearson correlation, are based only on the average of all values correlated. In contrast, our model is able to reliably predict time-specific associations between the two dynamic functions in neutrophils, respecting each time point of stimulation. According to our results, a number of cytokines could be fitted to the different types of neutrophil granules. These granules have been characterized to be mobilized towards the plasma membrane in a hierarchically and more precisely reverse order to their formation according to the* formed-first-released-last model* [2]. Our model illustrates the fact that neutrophil-derived cytokines and granules are released in a hierarchical sequence in accordance with their roles during the microbial elimination processes and inflammatory response (Figure 6(a)).

In this study, the linear fitting approach (i) gives us information about the concurrent behavior of cytokine secretion and degranulation upon inflammation, thus underlining the key role of both functions in the regulation of inflammatory responses and (ii) can represent an attractive method to investigate the possible mobilization or localization of cytokines in the different types of granules.

Given that cytokines can exert pleiotropic functions, some of them are probably localized in different types of granules as suggested by our model. In this sense, we found that IL8 correlated to secretory vesicles (Figure 6(a)), which are the most easily mobilized organelles in mature neutrophils [35]. Furthermore, IL8 was correlated to gelatinase and specific granules. These data support the observations of Pellmé et al. [2], who reported that IL8 is stored in cytoplasmic granules in resting peripheral blood neutrophils and, thus, can be rapidly mobilized and released by the cells.

IL8 can be secreted into the extracellular milieu from intracellular stores or by* de novo* synthesis via the classical secretory pathway. Two phases of secretion have been described: an early secretory phase which is directly induced by LPS and a late secretory phase which results from LPS-stimulated release of other proinflammatory mediators such as TNF*α* and IL1*β* [39]. The fact that IL8 is stored in different types of granules could allow its secretion over a large time interval.

Large amount of IL8 released could be explained by a positive feedback loop generated by MMP-9 on the IL8-induced neutrophil activity. Indeed, it has been previously reported that MMP-9 released from gelatinase granules is able to process IL8 [40] which is stored in the same granules as MMP-9 before secretion as shown by our linear fitting approach. IL8 cleaved by MMP-9 can enhance neutrophil degranulation [40], in comparison to nondegraded IL8, and thus increase the quantity of MMP-9 and IL8 released leading to an amplification of this system.

Our results also show a significant fitting between kinetics of IL6 secretion and the release of gelatinase granules. In line with this observation, Terebuh et al. [41] showed by immunohistochemical staining of neutrophils that IL6 might be localized in gelatinase granules and secretory vesicles.

Finally, in our model, IL1*β* could be fitted to gelatinase degranulation as previously postulated [17]. IL1*β* is secreted by a nonclassical secretory pathway (independent of endoplasmic reticulum and Golgi apparatus). Different release models have been suggested for IL1*β* [42] but the mechanisms associated are poorly understood and still controversially discussed. Our data indicate that the process of IL1*β* secretion involves a trafficking via granules which could be related to gelatinase granules.

In contrast to other granules, degranulation of azurophil granules seems unchanged by LPS suggesting that either (i) this granule type may require further cell activation to induce its mobilization towards the plasma membrane [43] or (ii) the upregulation of CD63 at the plasma membrane might not be significant enough to detect since azurophil granules are rather poor in receptors in contrast to secretory vesicles [35]. For this reason, no cytokine could probably be fitted to CD63 in human neutrophils.

In this view, late release of azurophil granule contents can be explained by the involvement of these proteins in neutrophil extracellular trap formation [43]. In this regard, the role of these granules during cytokine secretion appears very restricted. This assumption is supported by our results showing that azurophil granules are not able to translocate to the plasma membrane upon LPS stimulation in neutrophils.

## 5. Conclusion

Intracellular localization of cytokines in neutrophils remains largely elusive due to the fact that reliable staining for electron microscopy is facing challenges as the low intracellular amount of cytokines and other techniques used to document subcellular organelle location of cytokines have limited resolution (e.g., subcellular fractionation) [44].

In comparison to the modelling approach proposed by Rørvig et al. [45], which is based on proteomic and mRNA array data to predict localization of proteins in granules, our approach is complementary by including functional data analysis. Hence, the linear fitting between degranulation and cytokine release in LPS-treated neutrophils represents an attractive method to investigate the possible localization of cytokines in the different types of granules even if additional experiments are required to confirm the intracellular localization of cytokines. Furthermore, since our linear fitting approach has been adapted to investigate secretion kinetics, it can easily be extrapolated for the analysis of other short-time series deriving from other cell types, disease, or developmental states, for example, protein arrays or proteomics data.

Our linear fitting approach primarily constitutes a tool aiming to investigate regulatory mechanisms during neutrophil exocytosis but can also serve as basis to identify regulatory proteins by the supplementary analysis of proteins involved in exocytosis (e.g., SNARE and Rab proteins) and the construction of a dynamic regulatory network [46].

## Figures and Tables

**Table 1:** List of selected proinflammatory cytokines contributing to the development of chronic inflammatory disorders.

Cytokine	Cell recruitment	Chronic inflammatory disorders
TNF*α*	Monocytes, neutrophils, and dendritic cells	RA [13], IBD [18], A [19], and COPD [20]
IL1*α*	Neutrophils, T cells	RA [21], IBD [22]
IL1*β*	Neutrophils, thrombocytes, and T cells	RA [13]
IL6	Neutrophils, B cells	RA [13], A [23], and COPD [24]
IL12*β*	T cells	RA [25], IBD [26], and A [27]
CCL2	Monocytes, dendritic cells, and memory T cells	RA [21], A [28]
CCL3	Neutrophils, eosinophils, and basophils	RA [29], A [30]
CCL4	Monocytes, dendritic cells, NK cells, and T cells	RA [31], IBD [32], and A [30]
CCL5	Eosinophils, basophils, and T cells	RA [33], A [30]
IL8	Neutrophils, macrophages, and mast cells	RA [13], A [28], and IBD [34]

RA = rheumatoid arthritis, IBD = inflammatory bowel disease, A = atherosclerosis, and COPD = chronic obstructive pulmonary disease.

**(a):** 

Relative secretion	0 h LPS	2 h LPS	4 h LPS	6 h LPS	12 h LPS	24 h LPS
TNF*α*	1.28 ± 0.28	6.30 ± 1.81^*∗*^	24.82 ± 8.26	51.54 ± 14.79	36.70 ± 11.20^*∗∗∗*^	16.48 ± 11.35
IL1*α*	0.73 ± 0.38	1.43 ± 0.21	3.36 ± 1.92	6.84 ± 4.92	6.67 ± 3.51^*∗∗∗*^	14.70 ± 8.51^*∗*^
IL1*β*	0.69 ± 0.69	1.64 ± 0.2^*∗*^	4.87 ± 2.55	8.45 ± 5.89	7.89 ± 2.3^*∗*^	4.20 ± 3.46
IL6	0.93 ± 0.18	1.30 ± 0.37	34.35 ± 23.38^*∗*^	69.92 ± 50.91^*∗*^	185.53 ± 62.99^*∗∗∗*^	23.64 ± 10.51^*∗*^
IL12*β*	1.02 ± 0.06	0.88 ± 0.04	1.06 ± 0.09	1.16 ± 0.19	6.20 ± 3.57	1.08 ± 0.25
CCL2	0.97 ± 0.13	0.86 ± 0.17	1.11 ± 0.18	1.17 ± 0.36	6.43 ± 1.48^*∗∗*^	1.69 ± 0.66
CCL3	1.43 ± 1.43	6.72 ± 0.98^*∗∗*^	24.35 ± 4.51^*∗*^	66.06 ± 17.86^*∗*^	65.98 ± 24.41^*∗∗∗*^	11.05 ± 4.62
CCL4	1.06 ± 0.07	2.65 ± 0.76	8.10 ± 3.09^*∗*^	11.69 ± 5.84^*∗*^	69.14 ± 15.76^*∗∗∗*^	1.22 ± 0.72
CCL5	0.88 ± 0.05	1.07 ± 0.24	1.04 ± 0.34	0.89 ± 0.22	0.96 ± 0.18	1.05 ± 0.29
IL8	1.02 ± 0.12	4.89 ± 1.47^*∗*^	9.85 ± 2.31^*∗*^	15.84 ± 3.93^*∗*^	31.83 ± 10.15^*∗∗∗*^	27.00 ± 8.89^*∗*^

**(b):** 

Relative degranulation	0 h LPS	2 h LPS	4 h LPS	6 h LPS	12 h LPS	24 h LPS
CD63	1.00 ± 0.42	1.09 ± 0.33	0.97 ± 0.10	1.02 ± 0.12	0.88 ± 0.10	0.92 ± 0.12
CD15	1.00 ± 0.07	1.08 ± 0.14	1.26 ± 0.06^*∗∗*^	1.23 ± 0.07	1.56 ± 0.13^*∗∗*^	1.36 ± 0.16
CD66b	1.00 ± 0.06	1.14 ± 0.13	1.37 ± 0.12	1.72 ± 0.14^*∗∗∗*^	2.41 ± 0.13^*∗∗∗*^	1.87 ± 0.20^*∗∗*^
CD11b	1.00 ± 0.17	1.29 ± 0.14	1.62 ± 0.09^*∗∗*^	2.11 ± 0.21^*∗∗∗*^	2.57 ± 0.29^*∗∗∗*^	2.03 ± 0.34^*∗*^
CD13	1.00 ± 0.13	1.08 ± 0.10	1.14 ± 0.13	1.38 ± 0.14^*∗*^	1.94 ± 0.31^*∗*^	1.76 ± 0.32
CD14	1.00 ± 0.30	1.13 ± 0.33	1.31 ± 0.37	1.58 ± 0.44	1.23 ± 0.35	1.00 ± 0.31
CD18	1.00 ± 0.14	1.26 ± 0.26	1.23 ± 0.17	1.61 ± 0.3	2.22 ± 0.33^*∗∗*^	1.68 ± 0.27^*∗*^
CD45	1.00 ± 0.12	1.19 ± 0.15	1.39 ± 0.11^*∗*^	1.58 ± 0.16^*∗*^	2.18 ± 0.10^*∗∗∗∗*^	1.84 ± 0.22^*∗∗*^

## Abbreviations

CBA: Cytometric bead array
LPS: Bacterial lipopolysaccharide.
